# Allelic variation in the autotetraploid potato: genes involved in starch and steroidal glycoalkaloid metabolism as a case study

**DOI:** 10.1186/s12864-024-10186-5

**Published:** 2024-03-12

**Authors:** Hongbo Li, Matthijs Brouwer, Elena Del Pup, Natascha van Lieshout, Richard Finkers, Christian W. B. Bachem, Richard G. F. Visser

**Affiliations:** 1 Plant Breeding, Wageningen University & Research, P. O. Box 386, Wageningen, 6700 AJ the Netherlands; 2 Graduate School Experimental Plant Sciences, Wageningen University and Research, Wageningen, the Netherlands; 3 Shenzhen Branch, Guangdong Laboratory of Lingnan Modern Agriculture, Key Laboratory of Synthetic Biology, Ministry of Agriculture and Rural Affairs, Agricultural Genomics Institute at Shenzhen, Chinese Academy of Agricultural Sciences, Shenzhen, 518120 China; 4 SURFsara, Science Park 140, Amsterdam, 1098 XG the Netherlands; 5 Gennovation B.V, Agro Business Park 10, Wageningen, 6708 PW the Netherlands

**Keywords:** Potato, Allelic variation, Starch, Steroidal glycoalkaloid

## Abstract

**Background:**

Tuber starch and steroidal glycoalkaloid (SGA)-related traits have been consistently prioritized in potato breeding, while allelic variation pattern of genes that underlie these traits is less explored.

**Results:**

Here, we focused on the genes involved in two important metabolic pathways in the potato: starch metabolism and SGA biosynthesis. We identified 119 genes consisting of 81 involved in starch metabolism and 38 in the biosynthesis of steroidal glycoalkaloids, and discovered 96,166 allelic variants among 2,169 gene haplotypes in six autotetraploid potato genomes. Comparative analyses revealed an uneven distribution of allelic variants among gene haplotypes and that the vast majority of deleterious mutations in these genes are retained in heterozygous state in the autotetraploid potato genomes. Leveraging full-length cDNA sequencing data, we find that approximately 70% of haplotypes of the 119 genes are transcribable. Population genetic analyses identify starch and SGA biosynthetic genes that are potentially conserved or diverged between potato varieties with varying starch or SGA content.

**Conclusions:**

These results deepen the understanding of haplotypic diversity within functionally important genes in autotetraploid genomes and may facilitate functional characterization of genes or haplotypes contributing to traits related to starch and SGA in potato.

**Supplementary Information:**

The online version contains supplementary material available at 10.1186/s12864-024-10186-5.

## Background

Potato (*Solanum tuberosum* L.) is the most important non-cereal tuber crop and plays a critical role in global food and nutritional security, serving as a staple crop for more than a billion people worldwide [1–3]. Potato tubers are the harvested edible part and contain approximately 20% of dry matter, with most of it coming from starch [4]. Tuber starch content exhibits a great variation among potato varieties, ranging between 10% for table potatoes (fresh eating market) and up to 25% in potatoes bred for the starch processing industry [5]. Starch-related traits, including degree of phosphorylation, starch granule size, melting temperature, degree of branching of amylopectin and phosphate content of starch, can have profound impacts on the applications of potato starch, such as frying quality and the level of cold induced sweetening [6–8]. Beyond its use in food manufacturing, potato starch finds applications in other industries, including paper and textile manufacturing [9].

Potato tubers also contain several anti-nutrients that can be dangerous to humans if consumed in high amounts [3, 10, 11], one of which is steroidal glycoalkaloid (SGA), a secondary metabolite widely found in solanaceous plants including potato, tomato and eggplant [11]. Despite its importance in plant defense mechanisms, the content of SGA in potato tubers must be carefully controlled and has been the priority in food industries and breeding programs, especially during the introgression of desirable traits from wild relatives [10–12]. Phenotypes related with starch and SGAs are two pivotal categories that have been continuous selected during potato breeding: starch-related traits are central to breeding new varieties to meet the diverse preferences of various market types, while manipulation of tuber SGA content must be deployed to ensure that it falls into the acceptable and safe levels. Insights into their metabolic pathways and genetic regulation networks are thus essential to facilitate potato breeding.

In recent years, there have been significant advances in understanding the biosynthesis pathways of both starch and SGAs, with several key enzymes being functionally validated in model species such as Arabidopsis and tomato [10, 11, 13–17]. In potato tubers, starch synthesis occurs exclusively in the amyloplast, a specialized starch-accumulating plastid in heterotrophic tissues [18]. Sucrose acts as the glucosyl donor in tuber starch biosynthesis, which is transported from leaf tissues and converted to glucose-6-phosphate (G6P) in the cytosol of tuber cells [19]. Once inside the amyloplast, G6P is synthesized to ADP-glucose via the catalysis of phosphoglucomutase (PGM) and ADP-glucose pyrophosphorylase (AGPase). Amylopectin is next synthesized by starch synthases (SS) and starch branching enzymes (SBE), while amylose is produced by granular-bound starch synthase (GBSS) [9, 20].

SGAs in potato tubers are mainly composed of α-solanine and α-chaconine, whose biosynthesis can be divided into three main steps. The first step is the synthesis of cholesterol, the commonly accepted precursor of SGA formation. This step involves the catalysis of Acetyl-CoA to mevalonate by 3-hydroxy-3-methylglutaryl coenzyme A reductase (HMGR) [21]. Mevalonate is then converted to squalene-2,3-epoxide mediated by squalene synthase (SQS) and squalene epoxide (SQE), which is then formed into cycloartenol, the precursor of cholesterol, by oxidosqualene cyclases [17, 22]. The second step is the conversion of cholesterol to solanidine, which is catalyzed by a series of *GLYCOALKALOID METABOLISM* (*GAME*) enzymes, some of which have been functionally characterized in tomato and potato [23, 24]. In the final step of SGA metabolism, solanidine is glycosylated mediated by a group of glycosyltransferases comprising solanidine galactosyltransferase (SGT1), solanidine glucosyltransferase (SGT2) and glycosterol rhamnosyltransferase (SGT3) to form α-solanine and α-chaconine [10, 25]. A recent study also showed that an APETALA2/Ethylene Response Factor (*GAME9*) regulates expression of genes involved in synthesis of SGAs from solanidine and upstream mevalonate pathway [26].

Understanding of these biosynthetic pathways and the characterization of key genes participating in critical catalysis steps have permitted precise modification of starch and SGA-related phenotypes via biotechnological approaches. For example, overexpression of a plastidic ATP/ADP transporter from *Arabidopsis* in potato led to 16–36% of increase of starch content compared to control tubers [27]. Transgenic potato tubers with reduced level of α-glucan, water dikinase (GWD) [28] protein inhibited cold-induced sweetening [7], which is the accumulation of reducing sugars fructose and glucose in tubers during cold storage that leads to undesired dark colors when being fried. Down-regulation of *GBSS* resulted in transgenic potato tubers with reduced amylose, which is preferred in industrial applications [29]. Transgenic potato lines carrying either *HMGR* or *SQS1* from *Solanum chacoense*, a wild potato producing high content of SGA, exhibited elevated SGA levels compared with untransformed controls [17]. Overexpression of the soybean *C24-methyltransferase type 1* (*SMT1*) gene in transgenic potato displayed decreased level of free cholesterol, the precursor of SGA synthesis, which led to 41% and 63% of reduce of SGA level in leaves and tubers, respectively [30]. Moreover, antisense transgenic potato lines of SGT1, SGT2 or SGT3 involved in biosynthesis of α-solanine or α-chaconine reduced the corresponding SGA level relative to that of the wild type [25, 31, 32]. These findings lay a solid foundation for practical breeding and genomics-based selection of starch and SGA-related traits.

To further advance the understanding of the genetic basis of starch and SGA-related traits and facilitate marker-assisted breeding in potato, ample studies on quantitative trait locus (QTL) mapping or association studies have been performed. Over the past 20 years, several independent studies have identified QTLs or association signals for tuber starch content on all 12 potato chromosomes using diploid and tetraploid populations [5, 8, 33–40]. Some of them also proposed potential candidate genes, such as *AGPase* [37] and the invertase locus *invGE/GF* [40]. Some starch-related traits have also been analyzed via forward genetic approaches, suggesting associations between two phosphorylases (StPho1a and StPho2) and starch characteristics such as gelling temperature, chipping color, starch granule size and phosphorylation level [8]. Similarly, allelic variants within GWD, SS and SBE have been reported to be correlated with starch phosphorylation [41, 42]. QTLs and allelic variations in candidate genes responsible for regulation of SGA content in potato leaves and tubers have been detected on chromosome I, II, IV, VI, VIII, XI and XII [12, 43–47]. One study indicated that genes within a QTL on chromosome VIII were co-expressed with the *GAME* genes, possibly regulating SGA metabolism in potato tubers [12]. However, these studies have largely concentrated on allelic variation within a narrow set of loci, either localized at QTL/association signal regions or selected according to known important genes; leaving the genetic variation among the whole set of genes involved in starch and SGA metabolism unexplored. Meanwhile, tetraploid varieties dominate the potato industry and market, but patterns of allelic variation at haplotypic level among autotetraploid potatoes are still elusive. In this study, we generated an inventory of genes involved in starch and SGA metabolism comprising 81 starch and 38 SGA genes and revealed the haplotype-based landscape of their allelic variations in autotetraploid potato. In the light of population-scale resequencing data, we identified potentially conversed steps of starch and SGA metabolism and proposed genes that might have been diverged between tetraploid varieties with diverse levels of tuber starch or SGA content. These results provide valuable resources for further deciphering the genetic basis of starch and SGA-related agronomic traits in potato.

## Results

### An inventory of genes involved in potato starch and SGA metabolism

To obtain a nearly complete category of genes that participate in starch biosynthesis and degradation, we collected reported starch genes in the potato reference genome DM1-3 516R44 (hereafter referred to as DM) [9], National Center for Biotechnology Information (NCBI) GenBank accessions, and *Arabidopsis* gene symbols, and aligned their peptide sequences against the amino acid sequences of predicted genes from 48 potato reference genomes [48–53]. After manual inspection, we finally generated an 81-gene inventory of starch metabolism with an average gene length of 7.5 kb in the DM v6.1 reference genome, with no gene absent in DM but present in other potato accessions or species (Table 1). These genes encode enzymes involved in starch synthesis from sucrose in the cytosol to amylopectin and amylose in chloroplasts or amyloplasts, as well as starch degradation, The inventory also included some sugar transporter genes responsible for transporting substance, including glucosyl donors and ATP, from the cytosol to plastids. Some starch genes tend to cluster together, such as those on chromosome 3 (*Soltu.DM.03G007710.1*, *Soltu.DM.03G007720.1* and *Soltu.DM.03G007760.1* encoding α-glucan phosphorylases) and chromosome 7 (*Soltu.DM.07G018100.2*, *Soltu.DM.07G018130.1* and *Soltu.DM.07G018140.1* encoding β-amylases; Table 1).

**Table 1:** Summary of genes involved in starch metabolism

Gene information					Number of haplotypes					
Gene ID	Description	Chromosome	Start	End	Altus	Atlantic	Avenger	Colomba	Castle Russet	Spunta
*Soltu.DM.01G008290.1*	Triose-phosphate/phosphate translocator-like (TPT-like)	chr01	10,708,501	10,718,106	4	4	4	4	3	2
*Soltu.DM.01G012610.1*	Phosphoglucoisomerase-like 2 (PGI-like2)	chr01	23,288,582	23,289,601	3	3	4	4	4	3
*Soltu.DM.01G022570.2*	Beta-amylase 9 (BAM9)	chr01	60,987,038	60,990,400	4	3	3	3	3	3
*Soltu.DM.01G024440.1*	ADP-glucose pyrophosphorylase large subunit 1 (AGPL1)	chr01	63,686,919	63,691,316	4	2	4	4	3	2
*Soltu.DM.01G028790.1*	UDP-glucose pyrophosphorylase 1 (UGPase1)	chr01	68,549,182	68,561,301	4	3	4	3	3	4
*Soltu.DM.01G033560.1*	Beta-amylase 7 (BAM7)	chr01	73,206,343	73,213,592	3	4	3	3	2	3
*Soltu.DM.01G045740.1*	Inorganic pyrophosphatase (PPase)	chr01	83,484,092	83,488,381	2	4	3	3	3	4
*Soltu.DM.01G049590.2*	ADP-glucose pyrophosphorylase large subunit 3 (AGPL3)	chr01	86,376,380	86,381,406	4	2	3	3	2	4
*Soltu.DM.02G000530.1*	Disproportionating enzyme 2 (DPE2)	chr02	5,127,210	5,136,783	3	3	4	2	2	4
*Soltu.DM.02G009320.1*	Alpha-amylase 3-like (AMY3-like)	chr02	24,230,843	24,247,840	4	3	4	4	1	3
*Soltu.DM.02G014060.1*	Starch Synthase IV (SS4)	chr02	28,677,633	28,687,809	3	4	4	3	3	4
*Soltu.DM.02G017070.1*	Alpha-glucan phosphorylase 2b (PHO2b)	chr02	31,566,378	31,580,504	4	4	3	3	2	3
*Soltu.DM.02G020170.2*	Starch Synthase III (SS3)	chr02	34,367,686	34,383,334	4	4	3	4	3	3
*Soltu.DM.02G020800.2*	Sucrose Synthase 7 (SuSy7)	chr02	34,941,207	34,945,820	3	4	4	3	3	2
*Soltu.DM.02G026040.1*	Glucose transporter (GLT1)	chr02	39,127,419	39,137,166	4	3	2	2	2	3
*Soltu.DM.02G027020.1*	Starch Synthase V (SS5)	chr02	39,843,381	39,853,760	4	4	4	2	3	3
*Soltu.DM.02G031690.1*	Starch Synthase II (SS2)	chr02	43,711,583	43,718,240	2	4	4	1	3	4
*Soltu.DM.03G007710.1#*	Alpha-glucan phosphorylase 1a (PHO1a)	chr03	16,812,806	16,815,457	2	3	3	1	3	2
*Soltu.DM.03G007720.1#*	Alpha-glucan phosphorylase 1a (PHO1a)	chr03	16,821,960	16,826,524	2	3	3	1	3	2
*Soltu.DM.03G007760.1#*	Alpha-glucan phosphorylase 1a (PHO1a)	chr03	16,844,962	16,849,934	2	4	3	1	3	2
*Soltu.DM.03G008980.1*	Vacuolar Glucose Transporter 3-like (VGT3-like)	chr03	22,446,339	22,451,156	2	3	4	1	3	2
*Soltu.DM.03G013410.1*	Alpha-amylase 1.2 (AMY1.2)	chr03	35,837,209	35,840,386	3	4	3	3	3	4
*Soltu.DM.03G016410.1*	Phosphoglucomutase 1 (PGM1)	chr03	40,597,232	40,603,913	4	4	3	1	3	4
*Soltu.DM.03G016420.1*	Phosphoglucomutase 1 (PGM1)	chr03	40,607,920	40,613,538	4	4	3	1	2	2
*Soltu.DM.03G019120.1*	Sucrose Synthase 6 (SuSy6)	chr03	43,855,702	43,859,908	2	4	4	3	3	4
*Soltu.DM.03G022350.3*	Starch Synthase I (SS1)	chr03	47,124,469	47,133,578	3	2	3	3	4	4
*Soltu.DM.03G024560.1*	Phosphoglucan phosphatase (SEX4)	chr03	49,749,724	49,759,630	3	3	3	3	2	4
*Soltu.DM.03G034530.1*	ATP-ADP antiporter 1 (NTT1)	chr03	57,928,152	57,931,886	4	3	3	3	3	3
*Soltu.DM.04G015010.1*	Phosphoglucomutase 2.1 (PGM2.1)	chr04	24,544,911	24,553,865	3	3	3	3	4	4
*Soltu.DM.04G022990.1*	Disproportionating enzyme 1 (DPE1)	chr04	52,145,325	52,152,692	2	3	3	2	2	2
*Soltu.DM.04G025730.2*	Maltose excess 1 (MEX1)	chr04	56,008,096	56,013,296	3	3	2	2	3	3
*Soltu.DM.04G030730.1*	Phosphoglucoisomerase (PGI)	chr04	62,172,118	62,197,558	3	4	3	3	4	4
*Soltu.DM.04G033700.1*	Alpha-amylase 1.1 (AMY1.1)	chr04	65,328,724	65,330,894	4	2	3	2	3	4
*Soltu.DM.04G037250.1*	Alpha-amylase 2 (AMY2)	chr04	68,257,647	68,262,571	4	3	3	3	3	4
*Soltu.DM.04G037620.2*	Branching enzyme III (SBE3)	chr04	68,508,497	68,516,457	4	4	4	2	3	4
*Soltu.DM.05G000570.1*	Alpha-glucan phosphorylase 1b (PHO1b)	chr05	446,581	452,742	2	4	4	4	2	3
*Soltu.DM.05G006330.1*	Alpha-amylase 3 (AMY3)	chr05	6,037,246	6,053,915	2	3	4	3	4	3
*Soltu.DM.05G009520.2*	Glucan water dikinase (GWD)	chr05	9,901,255	9,916,669	3	4	3	2	4	3
*Soltu.DM.05G013630.1*	Putative Phosphoglucomutase (pPGM)	chr05	20,203,159	20,205,773	4	4	2	3	3	4
*Soltu.DM.05G013640.1*	Putative Phosphoglucomutase (pPGM)	chr05	20,206,673	20,212,607	4	4	2	3	2	3
*Soltu.DM.05G018750.1*	Glucose-6-phosphate/phosphate translocator 2.1 (GPT2.1)	chr05	45,902,088	45,905,436	3	4	3	3	2	3
*Soltu.DM.05G024440.1*	UDP-glucose pyrophosphorylase 1 (UGPase1)	chr05	52,978,332	52,986,180	4	4	4	4	3	3
*Soltu.DM.06G000410.2*	Isoamylase 3 (ISA3)	chr06	700,273	715,327	2	4	4	2	3	4
*Soltu.DM.06G010090.1*	UDP-glucose pyrophosphorylase3 (UGPase3)	chr06	30,638,103	30,649,525	3	4	4	3	4	4
*Soltu.DM.06G010900.1*	Phosphoglucan phosphatase (like SEX four 2, LSF2)	chr06	32,685,220	32,688,115	4	3	3	3	2	3
*Soltu.DM.07G005540.1*	Isoamylase 1.1 (ISA1.1)	chr07	7,354,469	7,370,232	4	4	4	4	4	3
*Soltu.DM.07G010140.1*	ADP-glucose pyrophosphorylase large subunit 2 (AGPL2)	chr07	28,459,732	28,465,251	3	3	2	3	3	3
*Soltu.DM.07G013360.1*	Sucrose Synthase 1 (SuSy1)	chr07	42,392,785	42,396,434	4	3	3	3	4	3
*Soltu.DM.07G013370.8*	Sucrose Synthase 2 (SuSy2)	chr07	42,423,839	42,428,022	4	3	3	3	3	2
*Soltu.DM.07G013620.1*	Starch Synthase VI (SS6)	chr07	42,699,355	42,708,860	4	4	3	3	4	2
*Soltu.DM.07G013630.1*	Starch Synthase VI (SS6)	chr07	42,712,584	42,720,966	3	3	3	4	4	3
*Soltu.DM.07G017870.1*	Glucose-6-phosphate/phosphate translocator 2.2 (GPT2.2)	chr07	48,392,736	48,395,736	2	4	3	3	3	3
*Soltu.DM.07G018100.2*	Beta-amylase 6.1 (BAM6.1)	chr07	48,609,121	48,613,209	2	4	3	3	3	2
*Soltu.DM.07G018130.1*	Beta-amylase 6.2 (BAM6.2)	chr07	48,660,084	48,661,302	1	1	1	1	2	1
*Soltu.DM.07G018140.1*	Beta-amylase 6.3 (BAM6.3)	chr07	48,675,418	48,676,516	1	1	1	1	2	1
*Soltu.DM.07G022290.1*	ADP-glucose pyrophosphorylase small subunit 1.1 (AGPS1.1)	chr07	52,424,941	52,430,371	2	4	2	3	3	2
*Soltu.DM.07G025710.1*	Branching enzyme I.2 (SBE1.2)	chr07	55,386,292	55,405,743	2	3	3	2	3	3
*Soltu.DM.07G025810.1*	Glucose-6-phosphate/phosphate translocator 1.1 (GPT1.1)	chr07	55,457,471	55,462,445	2	4	2	2	3	2
*Soltu.DM.07G026510.1*	Branching enzyme I.1 (SBE1.1)	chr07	55,936,361	55,953,226	2	2	3	2	4	3
*Soltu.DM.08G001120.1*	Beta-amylase 3.2 (BAM3.2)	chr08	1,618,565	1,622,209	3	3	4	3	3	4
*Soltu.DM.08G003130.1*	Beta-amylase 2 (BAM2)	chr08	4,084,723	4,094,664	4	4	3	3	3	2
*Soltu.DM.08G006240.1*	ADP-glucose pyrophosphorylase small subunit 2 (AGPS2)	chr08	8,843,996	8,847,025	4	4	3	4	3	3
*Soltu.DM.08G023420.1*	Beta-amylase 3.1 (BAM3.1)	chr08	53,049,020	53,052,674	3	4	4	4	4	3
*Soltu.DM.08G029750.1*	Beta-amylase 4 (BAM4)	chr08	58,749,933	58,756,635	3	3	4	2	4	2
*Soltu.DM.08G030230.3*	Granule bound starch synthase 1 (GBSS1)	chr08	59,143,807	59,147,539	4	3	4	4	4	3
*Soltu.DM.09G004100.1*	Branching enzyme II (SBE2)	chr09	3,535,898	3,555,467	3	4	4	2	4	4
*Soltu.DM.09G011580.1*	Alpha-glucan phosphorylase 2a (PHO2a)	chr09	20,467,173	20,479,953	4	4	3	3	3	2
*Soltu.DM.09G019230.1*	Isoamylase 2 (ISA2)	chr09	53,267,937	53,271,759	2	3	4	3	3	4
*Soltu.DM.09G027770.1*	Beta-amylase 1 (BAM1)	chr09	63,673,425	63,676,408	3	4	4	2	3	2
*Soltu.DM.09G030970.1*	Phosphoglucan water dikinase (PWD)	chr09	66,767,299	66,779,235	2	3	4	2	3	3
*Soltu.DM.09G031820.1*	Sucrose Synthase 3 (SuSy3)	chr09	67,526,937	67,534,221	4	3	4	2	4	3
*Soltu.DM.10G004860.3*	Triose-phosphate/phosphate translocator (TPT)	chr10	4,225,775	4,230,495	2	4	3	3	2	1
*Soltu.DM.10G007060.1*	Isoamylase 1.2 (ISA1.2)	chr10	7,897,543	7,899,618	1	2	2	3	2	2
*Soltu.DM.10G013240.1*	Inorganic pyrophosphatase-like (PPase-like)	chr10	38,286,777	38,293,554	1	2	4	2	2	1
*Soltu.DM.11G001030.1*	UDP-glucose pyrophosphorylase 2 (UGPase2)	chr11	1,109,263	1,115,893	3	3	4	3	3	4
*Soltu.DM.11G004600.1*	Limit dextrinase (LDE)	chr11	4,596,995	4,611,918	4	4	3	4	4	4
*Soltu.DM.11G004900.2*	Phosphoglucan phosphatase (SEX4-like)	chr11	5,006,751	5,013,766	4	4	4	4	3	4
*Soltu.DM.12G010800.1*	ATP-ADP antiporter 2 (NTT2)	chr12	10,748,355	10,752,085	2	3	4	4	4	4
*Soltu.DM.12G016610.4*	Phosphoglucan phosphatase (like SEX four 1, LSF1)	chr12	32,942,348	32,949,965	3	3	4	3	3	3
*Soltu.DM.12G026390.1*	Sucrose Synthase 4 (SuSy4)	chr12	56,336,213	56,342,227	4	2	2	4	2	3
*Soltu.DM.12G028820.2*	ADP-glucose pyrophosphorylase small subunit 1.2 (AGPS1.2)	chr12	58,423,903	58,431,045	4	3	3	3	4	3

# According to a previous study [1], the PHO1a locus in the DMv6.1 reference genome was mis-annotated into three separate genes, possibly owing to sequencing errors

1. Sharma S, Friberg M, Vogel P, Turesson H, Olsson N, Andersson M, Hofvander P: Pho1a (plastid starch phosphorylase) is duplicated and essential for normal starch granule phenotype in tubers of Solanum tuberosum L. Front Plant Sci 2023;14:1220973

We investigated expression patterns of the 81 starch-related genes in seven tissues of DM using publicly available RNA-seq data [48]. The majority of genes are expressed in most tissues, except for *Soltu.DM.01G008290.1* (Triose-phosphate/phosphate translocator-like), *Soltu.DM.07G017870.1* (Glucose-6-phosphate translocator), *Soltu.DM.08G006240.1* (ADP-glucose pyrophosphorylase small subunit), *Soltu.DM.12G028820.2* (ADP-glucose pyrophosphorylase small subunit) and *Soltu.DM.01G012610.1* (Phosphoglucoisomerase-like), whose expression is barely detectable (Figure S1). While some genes are not expressed or display a low expression level in stolons and tubers, their expression in other tissues, especially leaves, is much higher. Examples of such genes include those encoding isoamylase, β-amylase and α-glucan phosphorylase, all of which are involved in starch degradation (Figure S1).

Based on available knowledge of SGA metabolism and isoprenoid biosynthesis [10, 11, 17, 23–26, 54, 55], we extracted corresponding NCBI RefSeq accessions or gene symbols from *Arabidopsis* or tomato and aligned their sequences to available potato genomes. We manually checked the results and identified 38 genes associated with SGA metabolism, with an average gene length of 5.1 kb. Besides enzymes involved in catalysis from Acetyl-CoA to α-solanine and α-chaconine, we also incorporated genes exhibiting regulatory functions, such as *GAME9*, and a key enzyme participating in a major branch of Cycloartenol metabolism (SMT1; Table 2). These SGA genes were found on all the 12 chromosomes, except for chromosome 9 and 11, and some genes formed clusters (Table 2). Expression atlas analysis identified some genes exhibiting tissue-specific expression patterns. Examples included *Soltu.DM.06G004470.1* (A cytochrome P450 enzyme, GAME4CH6), which only expresses in fruits, *Soltu.DM.04G019820.1* and *Soltu.DM.12G024150.1* (Two cycloartenol synthases, CAS1) that are exclusively expressed in leaves and *Soltu.DM.10G028430.1* and *Soltu.DM.10G016360.1* (Two squalene synthases, SQS) with expression only detected in roots and flowers, respectively (Figure S2). These results may guide functional characterization of potato starch and SGA genes in various tissues.

**Table 2:** Summary of genes involved in SGA biosynthesis

Gene information					Number of haplotypes					
Gene ID	Description	Chromosome	Start	End	Altus	Atlantic	Avenger	Colomba	Castle Russet	Spunta
*Soltu.DM.01G027110.2*	Sterol C24-Methyltransferase (SMT1)	chr01	66,862,283	66,868,892	2	3	4	3	3	3
*Soltu.DM.01G031000.2*	Ethylene-responsive factor (GAME9)	chr01	70,710,298	70,711,462	2	2	2	2	4	1
*Soltu.DM.01G045860.1*	Squalene epoxidase (SQE)	chr01	83,550,333	83,553,990	2	4	3	3	4	4
*Soltu.DM.01G050130.3*	Squalene synthase (SQS)	chr01	86,823,576	86,832,687	2	2	3	2	3	2
*Soltu.DM.01G051390.1*	Sterol C24-Methyltransferase (SMT1)	chr01	87,913,487	87,914,898	3	3	2	4	2	3
*Soltu.DM.02G004910.1*	3-hydroxy-3-methylglutaryl coenzyme-A reductase (HMG2)	chr02	17,738,149	17,740,939	4	4	4	3	3	3
*Soltu.DM.02G007130.1*	Delta(7)-sterol-c5(6)-desaturase (C5-SD)	chr02	21,165,424	21,167,882	2	3	3	4	1	4
*Soltu.DM.02G007460.3*	Squalene epoxidase (SQE)	chr02	21,640,596	21,645,433	2	4	3	4	3	4
*Soltu.DM.02G012480.1*	Sterol side chain reductase (SSR2)	chr02	27,221,284	27,231,507	3	3	3	3	3	3
*Soltu.DM.02G022190.1*	3-hydroxy-3-methylglutaryl coenzyme-A reductase (HMG1)	chr02	35,953,719	35,957,358	2	4	3	3	2	3
*Soltu.DM.02G026060.1*	Delta(7)-sterol-c5(6)-desaturase (C5-SD)	chr02	39,146,404	39,149,183	3	2	2	3	3	3
*Soltu.DM.02G026070.1*	Delta(7)-sterol-c5(6)-desaturase (C5-SD)	chr02	39,153,719	39,156,674	3	3	2	3	4	4
*Soltu.DM.03G003200.1*	3-hydroxy-3-methylglutaryl coenzyme-A reductase (HMG3)	chr03	3,105,233	3,109,013	2	3	2	3	4	3
*Soltu.DM.04G019820.1*	Cycloartenol synthase (CAS1)	chr04	45,889,860	45,900,069	3	3	4	3	2	3
*Soltu.DM.04G026280.1*	Cycloartenol synthase (CAS1)	chr04	56,624,506	56,641,891	1	3	2	3	3	3
*Soltu.DM.04G032150.1*	Squalene epoxidase (SQE)	chr04	63,771,283	63,775,215	3	4	2	2	4	4
*Soltu.DM.04G038040.1*	3-hydroxy-3-methylglutaryl coenzyme-A reductases (HMG1)	chr04	68,867,941	68,871,704	4	3	3	2	3	4
*Soltu.DM.05G004250.1*	Sterol C24-Methyltransferase (SMT1)	chr05	3,669,646	3,670,626	2	3	3	3	4	3
*Soltu.DM.05G019230.1*	Cycloartenol synthase (CAS1)	chr05	46,836,492	46,847,111	4	4	3	3	2	4
*Soltu.DM.06G004470.1*	Cytochrome P450 (GAME4CH6)	chr06	6,057,871	6,063,493	3	3	3	4	2	3
*Soltu.DM.06G018370.1*	Cytochrome P450 (GAME8a, PGA1)	chr06	44,819,947	44,822,047	2	3	4	3	4	4
*Soltu.DM.06G018380.1*	Cytochrome P450 (GAME8b, PGA1)	chr06	44,859,734	44,861,585	2	3	4	3	4	3
*Soltu.DM.07G013460.1*	Cycloartenol synthase (CAS1)	chr07	42,520,849	42,530,066	3	3	3	3	3	4
*Soltu.DM.07G014160.1*	Glycosyltransferase (SGT3)	chr07	43,432,520	43,434,341	3	3	3	4	4	3
*Soltu.DM.07G014170.1*	Dioxygenase/oxidoreductase (GAME11)	chr07	43,555,537	43,557,153	3	3	3	4	4	3
*Soltu.DM.07G014190.1*	Cytochrome P450 (GAME6, PGA2)	chr07	43,594,461	43,596,848	2	3	3	3	4	3
*Soltu.DM.07G014220.1*	Galactosyltransferase (SGT1)	chr07	43,662,699	43,664,480	2	3	3	3	4	3
*Soltu.DM.07G015140.1*	Lanostetrol synthase (LAS1)	chr07	44,944,885	44,952,998	3	3	3	3	4	4
*Soltu.DM.07G023660.1*	Cytochrome P450 (GAME7)	chr07	53,630,069	53,634,086	2	3	2	3	3	2
*Soltu.DM.08G022920.1*	Glucosyltransferase (SGT2)	chr08	52,338,169	52,340,193	3	2	2	4	3	3
*Soltu.DM.10G016360.1*	Squalene synthase (SQS)	chr10	45,903,711	45,909,486	3	4	2	3	3	4
*Soltu.DM.10G017720.1*	Sterol C24-Methyltransferase (SMT1)	chr10	48,158,106	48,159,595	1	3	3	2	2	2
*Soltu.DM.10G027190.1*	Sterol C24-Methyltransferase (SMT1)	chr10	58,477,207	58,488,692	3	3	4	3	4	3
*Soltu.DM.10G028420.2*	Squalene synthase (SQS)	chr10	59,419,149	59,429,651	2	4	4	2	4	2
*Soltu.DM.10G028430.1*	Squalene synthase (SQS)	chr10	59,432,798	59,437,514	2	4	2	2	3	3
*Soltu.DM.12G024040.1*	Cytochrome P450 (GAME4CH12)	chr12	53,896,395	53,904,071	2	4	3	4	3	3
*Soltu.DM.12G024050.1*	Aminotrasferase/transaminase (GAME12)	chr12	53,969,804	53,977,550	2	4	3	4	3	3
*Soltu.DM.12G024150.1*	Cycloartenol synthase (CAS1)	chr12	54,102,884	54,107,311	3	3	2	3	3	3

### Allelic variants in starch and SGA genes of tetraploid potato

**Fig. 1:**
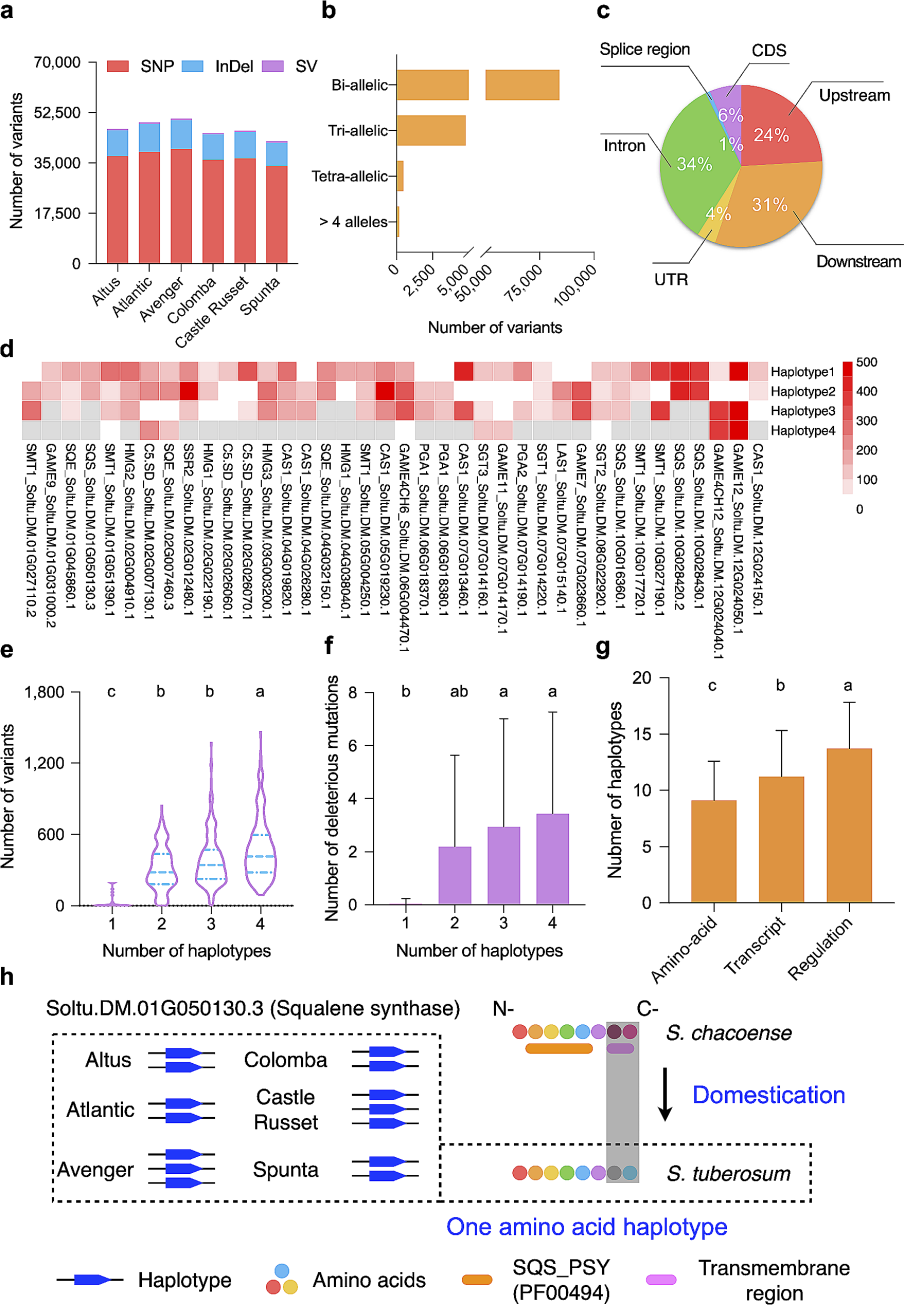
The landscape of haplotype-based allelic variation in starch and SGA genes among autotetraploid potato. **a**, Number of SNPs, InDels and SVs identified in the six tetraploid potato varieties. **b**, Number of variants containing different counts of alleles. A bi-allelic variation denotes that it only possesses one alternative allele and the reference allele among the six potato cultivars. **c**, Functional annotation of the identified variants as shown in the pie chart. **d**, Uneven distribution of allelic variation identified in 38 SGA genes among their haplotypes in the Colomba genome. Number of allelic variations identified in each haplotype is displayed in heat maps. Grey boxes indicate missing data (This gene does not contain this haplotype number). **e**, Violin plots depict number and distribution of genetic variants in genes carrying different numbers of haplotypes in each of the six potato genomes. The blue dashed lines indicate 75%, median and 25% quartiles. Multiple comparisons are performed using Kruskal-Wallis test with *α* = 0.001 **f**, Number of deleterious mutations in genes with one to four haplotypes in the six potato genomes. **g**, Number of types of haplotypes that can be defined in the six potato cultivars for starch and SGA genes. **h**, Domestication targeted on a gene encoding a squalene synthase (*Soltu.DM.01G050130.3*) may lead to a conserved amino acid haplotype structure among tetraploid potato cultivars, which is possibly essential in significant reduction of tuber SGA content in cultivated potato. The number of regulation haplotypes for *Soltu.DM.01G050130.3* varies from 2 to 4 across the six potato genomes (left panel), while only one amino-acid haplotype was identified (right panel). A high level of sequence divergence was observed around the predicted transmembrane region of this gene when comparing a wild potato species *S. chacoense* with cultivated potato. For **f** and **g**, data are presented as mean ± SD. One-way ANOVA and Turkey’s multiple comparisons with α = 0.01 are applied

Previous research reported six chromosome-scale haplotype-resolved autotetraploid potato genome assemblies (Altus, Atlantic, Avenger, Castle Russet, Colomba and Spunta) [56], which permits us to uncover the genetic diversity at the haplotype level within genes involved in starch and SGA pathways. We first extracted 2,169 gene haplotypes for the 119 starch and SGA genes from the six autotetraploid potato genomes, an average of three haplotypes per locus (Tables 1 and 2). A total of 73,228 single nucleotide polymorphisms (SNPs), 21,219 small insertions and deletions (InDels, ≤ 50 bp in length) and 1,719 structural variants (SVs, insertions and deletions > 50 bp in size) were then identified through pairwise alignment between sequences of each haplotype and the corresponding reference locus on DM. This long segment alignment-based variant calling approach preserves the haplotype information for each identified variant, while conventional methods relying on short read mapping can only partially capture haplotype information. We observe no significant difference among the six potatoes in terms of variant number (Fig. 1a; Tables S1 and S2). Around 94% of these variants are bi-allelic, with only 693 variants carrying more than three alleles among the six potato genomes (Fig. 1b). Most allelic variants were localized at intron regions, followed by 2-kb downstream and 2-kb upstream regions, and only 6% of variants impact coding sequences (CDS; Fig. 1c; Tables S3 and S4). The genetic variation identified herein provides a starting point for gaining access into haplotypic patterns in these functionally important genes.

### Haplotype-based characteristics of allelic variation

Since every variant in our allelic variation dataset is fully phased, we were able to investigate haplotypic features of variants in starch and SGA genes in autotetraploid potato genomes. We first examined whether the number of variants within different haplotypes for each gene is comparable. Intriguingly, we found that a large proportion of these genes displayed an uneven distribution of allelic variation among haplotypes. For instance, in 23 out of 38 SGA-related genes of Colomba, at least one haplotype contained markedly fewer number of variants compared to other haplotypes (Fig. 1d). Comparison of the number of genetic variations among genes containing different numbers of haplotypes in each of the six potato genomes (ranging from one to four) indicated that four-haplotype genes harbor the highest number of variants and those with only one haplotype carry drastically fewer variants (24 in average), while the difference between genes possessing two and three haplotypes was not significant (Kruskal-Wallis test, *α* = 0.001; Fig. 1e). Purge of deleterious mutations has been insufficient due to long-term clonal propagation and the lack of recombinants during potato breeding [57]. Therefore, potato genomes may still have a large number of unpurged deleterious variants. We identified 292 and 251 deleterious mutations in starch and SGA genes in the six potato genomes, respectively, and over 90% of these deleterious mutations were heterozygous in the corresponding genome. Additionally, we observed similar patterns in the number of deleterious substitutions among genes with different numbers of haplotypes, compared to the number of all allelic variants (Fig. 1f). These results suggest that homozygous loci in tetraploid potatoes may be less likely to tolerate the adverse impact of genomic and detrimental variants.

To explore haplotype patterns among the six-genome mini-collection, we classified haplotypes for each gene into three categories based on genomic location and functional impacts of allelic variants. The six genomes have the fewest number of amino acid haplotypes (averaging nine), which were defined as those sharing the identical peptide sequences, whereases the mean number of transcript haplotypes (defined by allelic variants localized from the 5’ to the 3’ untranslated regions) and regulation haplotypes (defined by variants in 2-kb upstream, genic and 2-kb downstream regions) was 11 and 14, respectively (Fig. 1g). Domestication has usually resulted in a substantial loss of genetic diversity in certain genomic regions of cultivated crop species [58]. We therefore investigated whether genes that have putatively undergone domestication had fewer haplotypes. We identified five starch genes and two SGA genes that resided in previously reported domestication sweeps [59], and found that the number of the three types of haplotypes in these domestication genes was comparable with other genes (Figure S3). Notably, we found that one of the domestication genes involved in SGA metabolism, *Soltu.DM.01G050130.3*, which encodes a squalene synthase (SQS), had a mere one amino acid haplotype among the six tetraploid potatoes (Fig. 1h). Around this locus, the density of allelic variants was slightly, but not significantly lower than that of other SGA genes (15.17 and 20.10 variants per kb, respectively, *p*-value = 0.0781 in two-tailed Student’s *t*-test). These results suggest that the constraint of its amino acid sequences might reflect important functions.

There are four putative SQS-encoding genes in the DM reference genome, and *Soltu.DM.01G050130.3* displays the highest expression level in all seven tissues (Figure S2). SQS catalyzes the formation of squalene, a precursor for sterol and SGA biosynthesis [60], and transgenic potato lines with SQS from the wild potato *S. chacoense* exhibited increased levels of tuber SGA content [17]. We applied pair-wise alignment between peptide sequences of the SQS ortholog in *S. chacoense* and *S. tuberosum* and observed extremely low identity within the C-terminal region, where a transmembrane region was predicted (Fig. 1h and Figure S4). These results suggest that domestication of potato might lead to the loss of normal function of this SQS through the impairment of transmembrane domain, which possibly resulted in potatoes with fewer squalene and thus low tuber SGA content. Artificial selection imposed on this genomic region might produce a conserved haplotype structure among modern potato varieties.

### Haplotype-based transcriptional landscape in starch and SGA genes of tetraploid potato

**Fig. 2:**
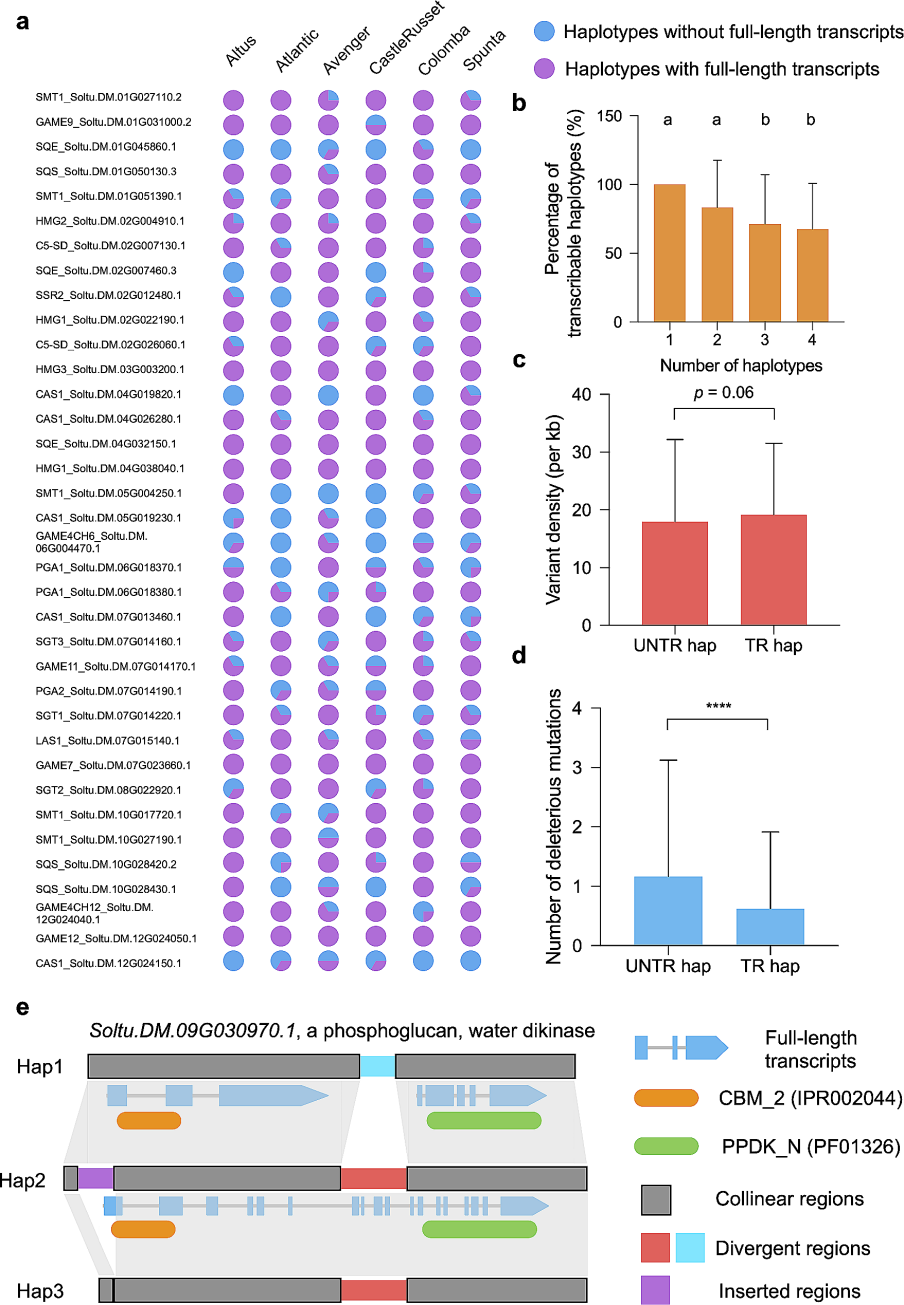
Patterns of transcribable and un-transcribable haplotypes unraveled in genes involved in starch and SGA metabolism among autotetraploid potato. **a**, Composition of transcribable and un-transcribable haplotypes in 36 SGA-related genes among six tetraploid potato cultivars illustrated by a 36 × 6 matrix of pie charts. **b**, Percentage of transcribable haplotypes in starch and SGA genes carrying one to four haplotypes in each of the six cultivars. One-way ANOVA and Turkey’s multiple comparisons with *α* = 0.01 are applied. **c**, Density of genetic variations (per one kilo base pairs) identified in un-transcribable haplotypes (UNTR hap) and transcribable haplotypes (TR hap). *P* value is calculated using two-tailed *t*-test. **d**, Number of deleterious mutations predicted in un-transcribable haplotypes (UNTR hap) and transcribable haplotypes (TR hap). **** *p* < 0.0001 in two-tailed Wilcoxon rank sum test. **e**, Three types of transcripts derived from a single locus carrying three haplotypes as exemplified by *Soltu.DM.09G030970.1*, a phosphoglucan, water dikinase. The reference haplotype is Hap2 with a transcript whose translated peptides containing two predicted functional domains CMB_2 and PPDK_N. A large deletion present in Hap3 probably leads to the non-transcript outcome. The complete transcript is divided into two independent properly aligned full-length transcripts in Hap 1 possibly due to a substitution of a divergent region. Data are presented as mean ± SD in **b**-**d**

Modern cultivated potato has four sets of homologous chromosomes (haplotypes), while the number of transcribable haplotypes within a given locus is largely unexplored. To better understand this, we downloaded previously released Pacific Biosciences (PacBio) isoform sequencing (Iso-Seq) data for Altus, Avenger, Colomba and Spunta and Oxford Nanopore technologies (ONT) full-length cDNA sequencing reads for Atlantic and Castle Russet [56] (from leaves and tubers). We then generated full-length non-chimeric (FLNC) reads and then mapped them against the 2,169 haplotypes of the 81 starch and 38 SGA genes. After manual inspection of alignments from each haplotype, we found that 68.82% (1,021 of 1,488) of starch-gene haplotypes and 65.35% (445 of 681) of haplotypes of SGA genes contained properly mapped full-length transcripts, indicating that these haplotypes are transcribable. Among the six potato cultivars, an average of 56.67% of starch genes and 51.85% of SGA genes contained haplotypes that are all transcribable, whereas 32.67% and 36.57% of starch and SGA genes display a mixed composition of transcribable and un-transcribable haplotypes, respectively (Figure S5; Fig. 2a; Tables S5 and S6). We also observed that genes containing fewer haplotypes had higher proportions of transcribable haplotypes, with single-haplotype genes being all transcribable, while 67.44% of haplotypes of four-haplotype genes had properly mapped transcripts (Fig. 2b). We further investigated patterns of genetic variations within these haplotypes and found that the density of all identified allelic variations within transcribable and un-transcribable haplotypes was similar, both of which possessed an average of ~ 20 variants per kb (Fig. 2c). The number of SVs identified in these two classes of haplotypes was also comparable (Figure S6). Intriguingly, we found that the number of deleterious mutations predicted in transcribable haplotypes was significantly lower than that in their un-transcribable counterparts (Fig. 2d; Wilcoxon rank sum test, *p* < 0.0001). These results suggest that transcribable haplotypes may have undergone purifying selection.

Heterozygous potato exhibits a high degree of intra-haplotype divergence, as exemplified by over 2% in the diploid potato accession RH89-039-16 [52], which might contribute to high levels of structural differences among transcripts derived from different haplotypes. Notably, a total of 19 (25.3%) starch genes and six (16.2%) SGA-related genes carrying multiple haplotypes displayed a diverse transcript architecture in at least one of the six autotetraploid potatoes. Among these genes, *Soltu.DM.09G030970.1*, which encodes a Phosphoglucan, water dikinase (PWD) and plays a critical role in starch phosphorylation and degradation [61], carries three diverse haplotypes in the Spunta genome. Full-length transcript alignments indicated that two short transcripts with several isoforms were mapped (mapping quality = 40) to haplotype 1 (Hap1), with no clip at both sides, and one long transcript was aligned to haplotype 2 (Hap2). However, no properly mapped transcript was identified on haplotype 3 (Hap3; Figure S7). To further tap into “three haplotypes, three transcripts” in this locus, we aligned sequences of these three haplotypes and identified a divergent region (low sequence similarity) between Hap1 (890 bp) and Hap2 (2,313 bp) and a 1,793-bp deletion in Hap3 compared with Hap2. The divergent region was localized inside the transcript of Hap2, thereby possibly resulting in structural changes in transcripts of Hap1. Notably, functional domains CBM_2 (starch binding domain) and PPDK_N (nucleotide binding domain) were present in the two separated transcripts from Hap1, respectively. The 1,793-bp deletion removed a part of the first exon of transcript of Hap2, which may render Hap3 un-transcribable (Fig. 2e). Therefore, we postulate that only transcripts from Hap2 may possess the normal function of PWD in Spunta. These results highlight the complexity of transcriptional landscape in autotetraploid species such as potato.

### Potentially conserved or diverged genes involved in starch and SGA metabolism

**Fig. 3:**
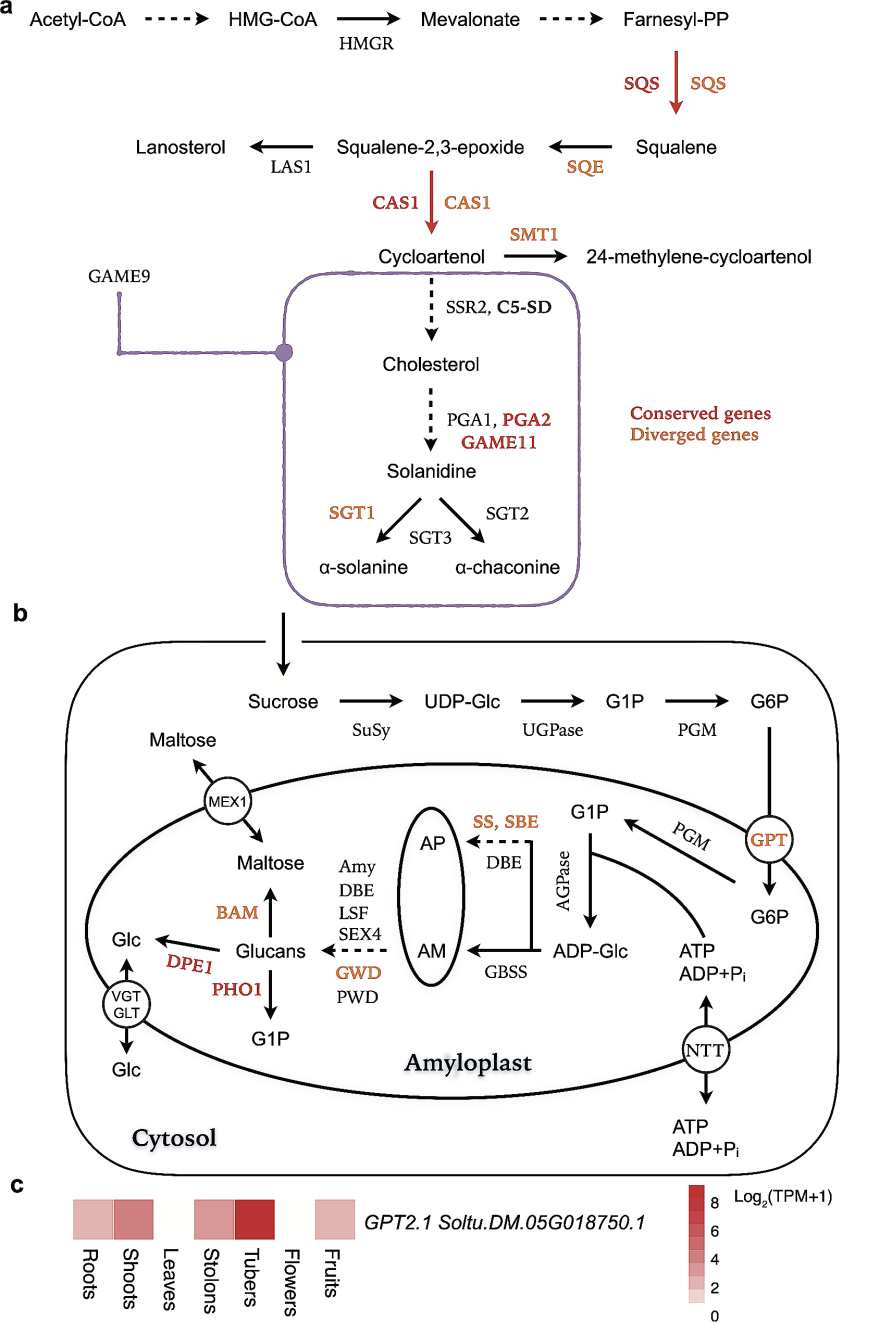
Potentially functionally important genes involved in SGA and starch metabolism. **a**, Five putatively conserved genes (red color) and five diverged (orange color) genes between cultivars with high and low total SGA content in the proposed SGA biosynthesis pathway. C5-SD, delta(7)-sterol-c5(6)-desaturase; CAS1, cycloartenol synthase; GAME9, GLYCOALKALOID METABOLISM 9; HMGR, 3-hydroxy-3-methylglutaryl coenzyme-A reductase; HMG-CoA, 3-hydroxy-3-methylglutaryl-coenzyme A; LAS1, lanostetrol synthase; PGA1, POTATO GLYCOALKALOID BIOSYNTHESIS 1; PGA2, POTATO GLYCOALKALOID BIOSYNTHESIS 2; SMT1, sterol C24-methyltransferase; SGT1, galactosyltransferase; SGT2, glucosyltransferase; SGT3, glycosyltransferase; SQE, squalene epoxide; SQS, squalene synthase; SSR2, sterol side chain reductase 2. **b**, Potentially conserved (red color) and diverged (orange color) genes between potato accessions bred for starch industry and other usages. The proposed starch biosynthesis and degradation pathway in potato tubers are depicted. ADP-Glc, ADP-glucose; AGPase, ADP-glucose pyrophosphorylase; AM, amylose; Amy, α-amylase; AP, amylopectin; BAM, β-amylase; DBE, starch branching enzyme; DPE, disproportionating enzyme; G1P, glucose 1-phosphate; G6P, glucose 6-phosphate; GBSS, granule-bound starch synthase; Glc, glucose; GLT, glucose transporter; GPT, glucose 6-phosphate/phosphate translocator; GWD, α-glucan, water dikinase; LSF, Like starch-excess Four; MEX, maltose transporter; NTT, nucleotide translocator; PGM, phosphoglucomutase; PHO, α-glucan phosphorylase; PWD, phosphoglucan, water dikinase; P_i_, inorganic pyrophosphate; SuSy, sucrose synthase; SS, starch synthase; SBE, starch branching enzymes; SEX4, starch excess 4; UGPase, UDP-glucose pyrophosphorylase; UDP-Glc, UDP-glucose; VGT, vacuolar glucose transporter. **c**, Expression pattern of *Soltu.DM.05G018750.1* (*GPT2.1*) on a log_2_ scale in seven tissues of DM. In **a** and **b**, dashed arrows indicate steps containing multiple catalytic reactions

Numerous genes have been reported to be involved in pathways of starch and SGA biosynthesis and degradation in potato; however, which genes are evolutionary conserved or diverged among the modern commercial potato varieties is less explored. To identify potentially conserved starch and SGA genes in potato genomes, we identified 109,883 genetic variants within the genic regions and 2-kb up- and down- streams of the 81 starch genes and 38 SGA genes, using genome-wide resequencing data from 137 autotetraploid potato varieties (Table S7). These genes possessed nucleotide diversity (π) ranging from 2.39 × 10^− 4^ to 8.60 × 10^− 3^ (Tables S8 and S9). We then proposed five SGA and five starch genes that displayed the lowest level of genomic diversity, which might be functionally constrained among the 137 tetraploid potato varieties (Fig. 3a,b; Table 3). Intriguingly, four (*Soltu.DM.03G007710.1*, *Soltu.DM.03G007720.1* and *Soltu.DM.03G007760.1* encode α-glucan phosphorylase 1a [PHO1a] and *Soltu.DM.04G022990.1* encodes a disproportionating enzyme [DPE1]) out of the five conserved genes are involved in starch degradation in the amyloplast (Fig. 3b).

**Table 3:** Genes involved in starch and SGA metabolism that are putatively conserved

Category	Gene ID	Description	Chromosome	Start	End
Starch	*Soltu.DM.03G007760.1*	Alpha-glucan phosphorylase 1a (PHO1a)	chr03	16,844,962	16,849,934
Starch	*Soltu.DM.10G013240.1*	Inorganic pyrophosphatase-like (PPase-like)	chr10	38,286,777	38,293,554
Starch	*Soltu.DM.03G007720.1*	Alpha-glucan phosphorylase 1a (PHO1a)	chr03	16,821,960	16,826,524
Starch	*Soltu.DM.04G022990.1*	Disproportionating enzyme 1 (DPE1)	chr04	52,145,325	52,152,692
Starch	*Soltu.DM.03G007710.1*	Alpha-glucan phosphorylase 1a (PHO1a)	chr03	16,812,806	16,815,457
SGA	*Soltu.DM.07G014190.1*	Cytochrome P450 (GAME6, PGA2)	chr07	43,594,461	43,596,848
SGA	*Soltu.DM.07G013460.1*	Cycloartenol synthase (CAS1)	chr07	42,520,849	42,530,066
SGA	*Soltu.DM.07G014170.1*	Dioxygenase/oxidoreductase (GAME11)	chr07	43,555,537	43,557,153
SGA	*Soltu.DM.04G026280.1*	Cycloartenol synthase (CAS1)	chr04	56,624,506	56,641,891
SGA	*Soltu.DM.10G016360.1*	Squalene synthase (SQS)	chr10	45,903,711	45,909,486

The 137 potato varieties used in this study were divided into high and low SGA groups based on their total SGA content (Table S7). Leveraging the measurement of neutrality selection (Tajima’s *D*) and population divergence, fixation index (*F*_ST_; Table S8), we identified five possible genes that might have been highly divergent between these two groups. These genes are *Soltu.DM.01G045860.1* (Squalene epoxide, SQE), *Soltu.DM.07G014220.1* (Galactosyltransferase, SGT1), *Soltu.DM.04G019820.1* (Cycloartenol synthase, CAS1), *Soltu.DM.01G027110.2* (Sterol C24-Methyltransferase, SMT1) and *Soltu.DM.01G050130.3* (Squalene synthase, SQS; Fig. 3a; Table 4). Note that CAS1 (*Soltu.DM.04G019820.1*) and SQS (*Soltu.DM.01G050130.3*) identified here were different paralogs of those that were putatively conserved genes (*Soltu.DM.07G013460.1* and *Soltu.DM.10G016360.1*, respectively). These genes mainly participate in the catalytic processes prior to cholesterol biosynthesis (Fig. 3a).

**Table 4:** Genes involved in SGA metabolism that are putatively diverged between varieties with high and low total SGA content

Gene ID	Description	Chromosome	Start	End
*Soltu.DM.01G045860.1*	Squalene epoxidase (SQE)	chr01	83,550,333	83,553,990
*Soltu.DM.07G014220.1*	Galactosyltransferase (SGT1)	chr07	43,662,699	43,664,480
*Soltu.DM.04G019820.1*	Cycloartenol synthase (CAS1)	chr04	45,889,860	45,900,069
*Soltu.DM.01G027110.2*	Sterol C24-Methyltransferase (SMT1)	chr01	66,862,283	66,868,892
*Soltu.DM.01G050130.3*	Squalene synthase (SQS)	chr01	86,823,576	86,832,687

We categorized the 137 potato varieties into two groups: one comprises 21 cultivars bred for the starch industry, representing a high starch content, and the other has the remaining 116 varieties with a diverse range of starch content (Table S7). Using Tajima’s *D* and *F*_ST_ statistics (Table S9), we also identified five possible genes that were greatly differentiated between these two groups: *Soltu.DM.05G009520.2* (Glucan water dikinase, GWD), *Soltu.DM.05G018750.1* (Glucose-6-phosphate/phosphate translocator 2.1, GPT2.1), *Soltu.DM.02G027020.1* (Starch Synthase V, SS5), *Soltu.DM.01G033560.1* (β-amylase 7, BAM7) and *Soltu.DM.09G004100.1* (Branching enzyme II, SBE2; Fig. 3b; Table 5). Intriguingly, one of these genes, *Soltu.DM.05G018750.1* (*GPT2.1*), which encodes a glucose-6-phosphate/phosphate translocator that transfers glucose-6-phosphates (G6P) from the cytosol to the amyloplast, was predominately expressed in potato tubers indicated by RNA-seq data of the potato DM (Fig. 3c). Previous studies have shown that knockout of GPT2 in *Arabidopsis* resulted in lower starch content [62], and associations were also observed between senescent sweetening and decrease of GPT2 transcript in potato tubers [63], suggesting possible roles of *GPT2.1* in starch metabolism of potato.

**Table 5:** Genes involved in starch metabolism that are putatively diverged between varieties bred for the starch processing industry and others

Gene ID	Description	Chromosome	Start	End
*Soltu.DM.05G009520.2*	Glucan water dikinase (GWD)	chr05	9,901,255	9,916,669
*Soltu.DM.05G018750.1*	Glucose-6-phosphate/phosphate translocator 2.1 (GPT2.1)	chr05	45,902,088	45,905,436
*Soltu.DM.02G027020.1*	Starch Synthase V (SS5)	chr02	39,843,381	39,853,760
*Soltu.DM.01G033560.1*	Beta-amylase 7 (BAM7)	chr01	73,206,343	73,213,592
*Soltu.DM.09G004100.1*	Branching enzyme II (SBE2)	chr09	3,535,898	3,555,467

We next identified 576 genetic variants within the genic region of *GPT2.1* and its 2-kb upstream and downstream regions, including nine non-synonymous SNPs, two 3-bp in-frame deletions and one splice-donor SNP. Among them, two missense SNPs (SNP126, asparagine to histidine and SNP387, alanine to threonine) and the splice-donor SNP were localized at the triose-phosphate transporter (TFT) domain encoding region. The two non-synonymous substitutions occurred at conserved sites when aligning orthologs from nine species (Figure S8). We also observed a clear differentiated pattern between the starch varieties and varieties bred for other market niches in SNP126 and SNP387 (Figure S9), suggesting possible selection targeted on *GPT2.1*. Therefore, we speculate that this gene may play a role in the selection of potato varieties with different levels of tuber starch content as well as other tuber quality traits, while its function remained to be validated by experimental approaches.

## Discussion

A comprehensive identification of genes involved in a specific pathway is crucial to enhance our understanding of underlying mechanisms. A previous study reported 77 loci that participate in potato starch biosynthesis and degradation based on DM v4.03 reference genome and publicly available transcripts [9]. However, the assembly of DM v4.03 was built by Illumina short reads, which resulted in a considerable number of unfilled gaps, mis-assemblies, and incompletely assembled regions due to sequencing bias. These issues impeded complete and accurate prediction of protein-coding genes. In this study, we utilized the long read-based genome assembly of DM v6.1 with its improved gene prediction and identified 81 starch-related genes, some of which were not present in the DM v4.03 reference genome. Nevertheless, we also found that some of these genes in DM v6.1 displayed a different structure compared with corresponding loci in DM v4.03, possibly due to different gene prediction strategies employed in these genome projects. We also presented a catalog of 38 genes involved in SGA metabolism and regulation. These datasets provide valuable resources for in-depth functional investigation of starch and SGA-related genes and will facilitate an enhanced understanding of the complex regulation and metabolic network in these critical pathways in potato.

Throughout the long-term clonal propagation in potato breeding, accumulation of genetic variations has been rampant in the four homologous chromosomes of potato due to the lack of purging mechanisms such as selfing, which has led to extreme haplotypic differences in both diploid and tetraploid potatoes. Our results for starch and SGA metabolism genes revealed an uneven distribution of allelic variants among haplotypes, a prevalent pattern observed in all six autotetraploid potato genomes (Fig. 1d). Another interesting finding is the consistent presence of a haplotype that displays a high degree of sequence identity (> 99%) when compared to the corresponding locus on the DM reference genome. We thus speculate that haplotypes that are highly similar to their reference loci on DM might retain their normal functions, while functions of others carrying markedly higher number of variants might have been disrupted or even lost. This is further supported by our finding that genes containing only one haplotype carry significantly fewer variants and deleterious mutations (Fig. 1e,f).

Why do genes that have only one haplotype carry significantly fewer allelic variants when compared with the DM reference genome? Given our hypothesis that DM-like gene haplotypes can produce transcripts of normal functions, there may be strong selection pressure on these genes to maintain identical haplotypes on the four homologous chromosomes, thereby achieving four-fold dosage to exert their functions. Further studies could focus on these one-haplotype genes to examine whether reduced transcript expression leads to functional consequences.

The full-length transcript sequencing data empowered haplotype-sensitive alignment, providing a preliminary understanding of the transcriptional pattern in autotetraploid potato. We noticed that approximately 70% of haplotypes are putatively transcribable in starch and SGA genes, while all one-haplotype genes produce transcripts (Fig. 2b). This suggests that the accumulation of genetic variants, some of which may display detrimental effects, may have an impact on the transcription of gene haplotypes in autotetraploid potato, in concordance with our finding that transcribable haplotypes have significantly fewer predicted deleterious mutations than do these un-transcribable haplotypes (Fig. 2d). Our results also indicate that transcribable haplotypes can carry a high number of allelic variations (Fig. 2c). Nevertheless, these haplotypes, though they are transcribable, might not produce transcripts exerting normal functions, and their expression abundance remained opaque owing to the inability to quantify transcripts when using only full-length transcript sequencing reads. This necessitates further investigation with the aid of additional omics data. We also identified several loci in which haplotypes generate transcripts with distinct structure. Whether these transcripts retain their normal functions may also be examined in future studies.

Population genomics enables the identification of potentially functionally important genes involved in SGA and starch metabolisms. Intriguingly, *Soltu.DM.01G050130.3*, a gene that may have undergone domestication encoding a squalene synthase, was considered as a diverged gene between potato varieties with high and low total SGA content (Table 4). However, we also found that this gene was “conserved” in the six autotetraploid potatoes (Altus, Atlantic, Avenger, Colomba, Castle Russet and Spunta, all have relatively low SGA content), with only one amino-acid haplotype being identified (Fig. 1h). Therefore, *Soltu.DM.01G050130.3* could possibly serve as a promising candidate for modulating SGA content in some potato varieties exhibiting high levels of total SGA (e.g., Festien, 766.20 and Astarte, 309.02), as its critical role in SGA biosynthesis has been validated in a previous study [17]. Further studies could investigate the expression level of *Soltu.DM.01G050130.3* in high-SGA content potato varieties and whether reduction of its expression results in decreases SGA content.

Our results suggest that a high proportion of conserved genes involved in the starch metabolism function in starch degradation steps (Table 3; Fig. 3b), while genes that may have diverged between starch and other varieties mostly participate in the formation of amylopectin and substrate transfer (Table 5; Fig. 3b). Notably, three out of four PHO1-encoding genes in potato, which catalyzes glucose 1-phosphate (G1P) from Glucan (one step in starch degradation), were considered conserved, all encoding PHO1a and being highly expressed in all seven tissues. Conversely, the remaining *PHO1b* gene was not expressed in stolons and tubers, suggesting that the three *PHO1a* genes may be important in tuber starch degradation. Given that G1P is a precursor of starch, fine tuning of expression levels and enzyme activities of these *PHO1a* may have the potential to regulate starch yield in potato tubers [64]. Further research will be necessary to explore the functional implications of these observations.

We identified that *GPT2.1*, functioning in the transfer of G6P from the cytosol to the amyloplast, showed relatively high level of population differentiation between starch and other varieties, which may have been under positive selection. This finding makes *GPT2.1* a promising candidate for further functional characterization.

## Conclusions

We discovered 96,166 allelic variants among 81 genes involved in starch metabolism and 38 genes that participated in SGA biosynthesis, from six haplotyped-resolved autotetraploid potato genomes. Comparative analysis unveiled an uneven distribution of allelic variants among the gene haplotypes, with the majority of deleterious mutations observed in a heterozygous state within the autotetraploid potato genomes. We uncovered that approximately 70% of the haplotypes for the 119 genes were transcribable, based on full-length cDNA sequencing data. Furthermore, through population genetic analysis, we identified specific starch and SGA biosynthetic genes that potentially exhibit conservation or divergence patterns among potato varieties with contrasting levels of starch or SGA content. Our analyses shed light on allelic diversity of genes involved in starch and SGA metabolism and provide useful information for further screening of potential candidate genes associated with starch and SGA-related agronomic traits.

## Methods

### Identification of genes involved in starch metabolism in potato

Putative genes participating in starch metabolism in potato leaves and tubers were extracted according to candidate loci in DM1-3 516R44 (hereafter DM) v4.03, reported *Arabidopsis* starch genes and National Center for Biotechnology Information (NCBI) RefSeq/GenBank accessions described in [9]. Amino acid sequences of these genes were aligned to the DM v6.1 representative gene models [65] to determine their orthologs by BLASTp search (v2.8.1+) [66]. To eliminate potential effects of reference bias when using DM as the single reference, we also aligned amino acid sequences of these genes to other available potato reference genomes including the 44 diploid potato assemblies incorporated in a potato pan-genome study [53], RH89-039-16 [52], Solyntus, [51] *Solanum commersonii* and *Solanum chacoense* [49, 50], and found that three *Arabidopsis* gene AT4G24450, AT2G21590 and AT5G17523 could not be assigned to proper orthologous genes in these potato genomes, which indicated that these three genes are indeed absent in potato in the light of currently available reference genomes. Results for each locus were manually inspected to ensure the consistency between the two versions (v4.03 and v6.1) of genome assemblies and gene prediction. The sequence and gene prediction information on DM v6.1 were extracted as the final starch reference gene category, which is comprised of 81 genes encoding enzymes involved in starch biosynthesis.

### Identification of genes involved in steroidal glycoalkaloids metabolism in potato

The schematic depiction of steroidal glycoalkaloid (SGA) metabolism pathway described in [59] were used as a starting point to identify SGA metabolism related genes in potato. Note that genes that participate in brassinosteroid and phytosterol biosynthesis were excluded from this study. The NCBI RefSeq ID of known genes involved in SGA biosynthesis were obtained from Table S7 in [11]. Gene symbols of a series of *GLYCOALKALOID METABOLISM* (*GAME*) genes in tomato reference genome “Heinz1706” [67] were extracted from [23] and the locus name of *GAME9* in tomato was obtained from [26]. Gene models in DM v4.03 of *Sterol Side Chain Reductase 2* (*SSR2*) were obtained from [55]. Amino-acid sequences of these loci were downloaded from NCBI, Sol Genomics Network (https://solgenomics.net/organism/solanum_lycopersicum/genome) and Spud DB (http://solanaceae.plantbiology.msu.edu/) and were aligned against peptide sequences of predicted gene models in available potato reference genomes using BLASTp (v2.8.1+) [66]. The results were manually inspected to determine the ortholog and paralog genes in potato. All these genes could be properly aligned to DM v6.1 gene models. To resolve the issue of multiple hits with similar E-value or bit scores, we also checked the functional annotation of genes falling into these BLAST hits and only retained those with expected functions annotated, which led to a reference inventory containing 38 SGA metabolism genes.

### Expression patterns of starch and SGA genes in DM

Transcriptome sequencing reads of DM from a range of tissues were downloaded from NCBI short read archive under accession number SRA030516. Data from similar tissues were merged for further processing, which finally retained roots, shoots, leaves, stolons, tubers, flowers and fruits. RNA-seq reads were mapped to the DM v6.1 reference genome using HISAT2 (version 2.0.4) [68] with default parameters. StringTie (v2.1.5b) [69] was next used to apply genome-guided transcript assembly and estimation of expression levels of annotated genes in terms of transcripts per million (TPM), enabling the “-e -G” parameters.

### Extraction of haplotypes in the phased genomes of six tetraploid potato varieties

Haplotype-resolved genome assemblies of the six potato varieties [56] (Altus, Atlantic, Avenger, Colomba, Castle Russet and Spunta) enable direct extraction of gene haplotypes. Genic and 2-kb upstream and downstream (2-kb from 5’ or 3’ UTR) sequences of each gene from DM v6.1 in the target pathways was first aligned to each of the six genomes using the nucmer program within the MUMmer software package (v4.0.0rc1) [70] with “-maxmatch” parameter. The alignments were then filtered using the delta-filter program with “-r” parameter to obtain each position of each reference to its best hit in the query, which retained similar haplotypic alignments. Alignments with identity < 90% and block length < 100 bp were also filtered out. The results were then manually processed to extract the corresponding genomic coordinates of each gene in the six genomes.

### Identification of allelic variants among DM and the six potato varieties in genes involved in starch and SGA metabolism

Each sequence of potential haplotypes of starch and SGA pathway genes extracted from the six tetraploid genomes was aligned to its corresponding reference gene using the nucmer program incorporated in the MUMmer software package (v4.0.0rc1) [70] with “-maxmatch” parameter. Alignments were then processed by the delta-filter program with “-1” parameter to retain one-to-one best alignments only. We found that some alignments would be erroneously “cleaned” possibly due to bugs within the delta-filter program. To rescue such results, we manually checked each of the resultant files and modified the original nucmer alignments to meet the filtering criteria. The filtered alignment results in “delta” format were then passed to the delta2vcf program to extract within-block genetic variants comprising SNPs and InDels. Some alignments showed high levels of sequence divergence in terms of large insertions or deletions within the gene sequences, which could lead to loss of large sizes of coding regions of a certain gene. To integrate such information into our allelic variation dataset, we used the show-diff program to output potential structural differences from the filtered alignments and applied our in-house scripts to extract corresponding breakpoints in both reference and query sequences. Note that only events reported in show-diff outputs marked with “BRK” (inserted sequences from the begin or end of the reference) or “GAP” (namely, alignment gaps between two mutually consistent alignments) were considered. Those with inserted or deleted sequences containing too many assembled gaps (> 20%) were removed. All the identified variations were merged and converted into a file in VCF format.

For each reference gene in a given potato cultivar (Altus, Atlantic, Avenger, Colomba, Castle Russet and Spunta), the identified variants for all potential haplotypes were merged using bcftools (v1.9) merge [71] with parameters “-0 --merge all”. We utilized the alignment coordinates obtained from the show-coords program to determine whether a variant is absent (due to local sequence deletion) or possessing the reference type of allele. If the variant coordinate showed good alignment coverage based on the show-coords output, the variant genotype was set to the reference type, which is in accordance with the inference of bcftools. Otherwise, it was considered as the missing genotype.

### Functional annotation of genetic variation and definition of three types of haplotypes

Putative function impacts of the identified allelic variations were predicted using SnpEff (v4.3t) [72] with default parameters. Based on the predictions, we divided haplotypes for a certain reference gene into three categories: amino acid haplotypes exhibiting no change of peptide sequences, transcript haplotypes whose genic sequences (from 5’ UTR to 3’ UTR) are identical and regulation haplotypes, which is the original class inferred from pairwise alignments that considers variants localized at 2-kb upstream, genic and 2-kb downstream regions.

### Prediction of deleterious mutations

A customized database for potato genome DM v6.1 was constructed using the officially recommended process according to SIFT 4G (sorting intolerant from tolerant for genomes) pipeline [73] and the VCF file for each reference gene was input to the SIFT 4G annotator to predict potential deleterious mutations based on the database, which is the situation that a non-synonymous substitution exhibiting the SIFT score ≤ 0.05.

### Identification of genes potentially involved in domestication

The reported domestication sweep regions on the DM reference genome v4.03 were first aligned to the DM reference genome v6.1, and genomic coordinates of each region were manually checked to extract the corresponding region in the v6.1 genome. Genomic positions of 81 starch and 38 SGA genes were then compared with these sweep regions and those showing overlaps were considered as putatively domestication-related genes. These genes comprise five starch genes (*Soltu.DM.07G026510.1, Soltu.DM.03G016410.1, Soltu.DM.03G016420.1, Soltu.DM.02G031690.1* and *Soltu.DM.09G031820.1*) and two SGA genes (*Soltu.DM.01G050130.3* and *Soltu.DM.07G014220.1*).

### Analysis of Pacific Biosciences isoform sequencing reads and Oxford Nanopore technologies full-length cDNA sequencing reads

Pacific Biosciences (PacBio) isoform sequencing (Iso-Seq) data for Altus, Avenger, Colomba and Spunta and Oxford Nanopore technologies (ONT) full-length cDNA sequencing reads for Atlantic and Castle Russet were collected from a previous study [56]. For all six cultivars, data from leaves and tubers were collected. The PacBio data were first preprocessed using CCS program powered by PacBio (https://github.com/PacificBiosciences/ccs) followed by primer removal and barcode demuliplexing using lima (https://github.com/PacificBiosciences/barcoding). Trimming of Poly(A) tails and elimination of concatemer in these reads were performed using the “refine” subcommand in isoseq program (https://github.com/PacificBiosciences/IsoSeq) and full-length non-chimeric (FLNC) reads were generated through “isoseq cluster”, in both of which default parameters were applied. The ONT data were preprocessed using pychopper software (https://github.com/nanoporetech/pychopper) to filter out reads with qualities < 7.0 and to produce FLNC transcript sequences. GMAP (version 2020-10-14) [74] was then applied to aligned these FLNC reads to each of the haplotypes with parameters: “--cross-species -n 0 -f samse -z auto” to enable the long-read spliced alignment mode. We used StringTie v2.1.5b [69] to assemble the mapped reads into potential transcripts. These transcripts were then aligned to each of the identified haplotype sequences of 81 starch and 38 SGA genes of the six potato cultivars using GMAP. Only unique alignments were kept and we excluded secondary alignments. A haplotype is considered to be transcribed if it has one or more properly mapped assembled transcripts. This step is manually performed because long-read alignment usually leads to truncated mapped results with large segments of clipping on both sides (associated with low mapping qualities), an issue that can hardly be resolved by automatic pipelines. Considering that long-read RNA sequencing technologies can only capture transcripts with relatively high abundance, we excluded genes in which full-length transcripts cannot be identified in all haplotypes in all the six cultivars, which resulted in 75 starch and 36 SGA genes used for downstream presentation and analysis.

We manually screened these alignments for the 75 starch and 36 SGA genes in the six potato genomes and found that 19 starch and six SGA-related multi-haplotype genes displayed diverse transcript architecture, which is the case that transcripts properly mapped to different haplotypes within a given locus show considerably structural changes (e.g., absence of mapped transcript in one or more haplotypes, lack of exons and different exon/intron architecture). These genes are: *Soltu.DM.01G024440.1, Soltu.DM.01G027110.2, Soltu.DM.01G028790.1, Soltu.DM.01G045740.1, Soltu.DM.02G009320.1, Soltu.DM.02G014060.1, Soltu.DM.02G020170.2, Soltu.DM.04G030730.1, Soltu.DM.04G037620.2, Soltu.DM.05G000570.1, Soltu.DM.06G010900.1, Soltu.DM.07G005540.1, Soltu.DM.07G013620.1, Soltu.DM.07G018100.2, Soltu.DM.08G003130.1, Soltu.DM.08G029750.1, Soltu.DM.09G011580.1, Soltu.DM.09G030970.1, Soltu.DM.11G004600.1, Soltu.DM.12G010800.1* for starch metabolism and *Soltu.DM.01G027110.2, Soltu.DM.02G026060.1, Soltu.DM.04G019820.1, Soltu.DM.07G015140.1, Soltu.DM.12G024040.1, Soltu.DM.12G024050.1* for SGA metabolism. The gene *Soltu.DM.09G030970.1* was selected as an example.

### Read mapping and variant calling

Quality control of Illumina resequencing raw reads from 137 tetraploid varieties (Table S7) was first applied using fastp (v0.20.0) [75] with default parameters, which removed low-quality reads (mean phred quality < 15), reads contain too many Ns (> 5), and low-complexity reads, as well as trimmed potential adapters. We then mapped these clean reads to the 81 starch and 38 SGA metabolism related genes by BWA-mem (0.7.17-r1188) [76] using “-R” parameter to include read group header line information. The resulting alignments in BAM format were sorted using SAMTools (v1.9) [76], followed by removal of potential PCR duplicates using “MarkDuplicates” function in Genome Analysis Toolkit (GATK, Version: v4.1.4) [77]. The “HaplotypeCaller” functionality in GATK was first deployed to call potential genetic variants for each sample, producing results in GVCF format that preserve both variant and non-variant site information. Parameters “--sample-ploidy 4 --emit-ref-confidence GVCF” were set to enable the tetraploid calling mode. Leveraging the GVCF files, population-scale variant joint-genotyping was next performed using the “GenotypeGVCFs” subcommand in GATK with parameters “--allow-old-rms-mapping-quality-annotation-data --sample-ploidy 4”, resulting in a single VCF file containing sites with at least one sample carrying a variant. We then applied a set of hard filters to remove low-quality genetic variants. For SNPs, variants exhibiting QD < 2.0 or MQ < 40.0 or FS > 60.0 or SOR > 3.0 or MQRankSum < -12.5 or ReadPosRankSum < -8.0 or GQ < 20.0 were removed. QD, variant quality (from the QUAL field) divided by the depth of samples; MQ, mapping qualities; FS, phred-scaled probability for a given site if the strand bias exists; SOR, a test similar to the symmetric odds ratio test to estimate the strand bias; MQRankSum, Z-scores from Wilcoxon rank sum test of read mapping qualities supporting the reference allele and the alternate allele; ReadPosRankSum, Z-scores from Wilcoxon rank sum test of read position bias of the reference allele and the alternate allele; GQ. genotyping qualities. Regarding small InDels, the same criteria applied on SNPs were exploited but we altered the threshold of FS and SOR to 200.0 and 10.0, respectively. The final VCF contained high-confidence genetic variations with allele dosage information preserved.

### Population genomic analyses

Nucleotide diversity in terms of pair-wise nucleotide differences (π) was computed using PopGenome (v2.7.5) [78] using the allele frequency information for each of the identified bi-allelic genetic variants. The sliding window approach was applied using a window size of 500 bp and a step size of 100 bp. For calculation of the neutrality selection index Tajima’s *D* and a measure of population differentiation, the fixation index (*F*_ST_), we divided the 137-variety potato group into two groups: one is the starch group containing 21 varieties bred solely for the starch industry; the other comprises the remaining 116 cultivars. Allelic frequencies of variants between these two groups were compared and *F*_ST_ was calculated via PopGenome. π, Tajima’s *D* and *F*_ST_ were reported as the mean value of all allelic variants within a given gene.

### Identification of potentially conserved and diverged genes

Genes that are potentially conserved were defined as those showing the bottom five lowest nucleotide diversity. The negative value of Tajima’s *D* means the abundance of rare alleles in the population, suggesting purging of deleterious mutations and occurrence of selective sweeps. This could lead to evolutionary conversed sequences, implying putative positive selection targeted on this gene. The positive Tajima’s *D* values suggest an excess of medium-frequency (common) alleles, in accordance with balancing selection or population contraction [79]. To identify putatively diverged starch genes between varieties from the starch group (21 varieties) and other market groups (116 varieties), and diverged SGA genes between cultivars with the highest total SGA content (> 100 mg/kg fresh weight, ten cultivars) and the lowest (< 50 mg/kg fresh weight, 12 varieties), we extracted corresponding genes showing the top ten *F*_ST_ values. Since we do not know whether a gene has undergone positive selection in either group (high/low starch and high/low SGA), we also identified genes showing the bottom ten lowest negative Tajima’s *D* values within either group. These two sets of genes were then intersected, and overlapping genes with the top five *F*_ST_ and the bottom five Tajima’s *D* values were regarded as potentially diverged genes.

### Electronic supplementary material

Below is the link to the electronic supplementary material.

Supplementary Material 1

Supplementary Material 2

## Data Availability

Raw Illumina sequencing reads have been deposited in NCBI sequence read archive (SRA; https://www.ncbi.nlm.nih.gov/sra/) under BioProject accession number PRJNA944441. PacBio Iso-Seq data for Altus, Avenger, Colomba and Spunta and ONT full-length cDNA sequencing reads for Atlantic and Castle Russet were downloaded from NCBI under BioProject accession number PRJNA718240. Custom scripts and codes used in this study are available at GitHub (https://github.com/HongboDoll/PathwayGeneVariation).
