# Supplementary material for: Allelic variation in the autotetraploid potato: genes involved in starch and steroidal glycoalkaloid metabolism as a case study
Source: BMC Genomics. 2024 Mar 12;25:274. doi: 10.1186/s12864-024-10186-5 (PMC10936075; doi:10.1186/s12864-024-10186-5)
Supplement: Supplementary file 2 — Supplementary Material 2 [file 12864_2024_10186_MOESM2_ESM.docx]

**Table of contents**

**Figure S1. Expression heat maps of starch related genes in roots, shoots, leaves, stolons, tubers, flowers and fruits of DM, in which expression levels are depicted as Log_2_(TPM+1).**

**Figure S2. Expression heat maps of SGA related genes in roots, shoots, leaves, stolons, tubers, flowers and fruits of DM, in which expression levels are depicted as Log_2_(TPM+1).**

**Figure S3. Number of three types of haplotypes that can be defined in the six potato cultivars for genes within or without regions of domestication sweeps.**

**Figure S4. Pair-wise alignment of amino acid sequences of the SQS gene in M6 (wild type, *S. chacoense*) and DM (domesticated type, *S. tuberosum*).** The transmembrane region is marked using a pink bar.

**Figure S5. Overview of percentage of transcribable and un-transcribable haplotypes in 75 starch-related genes among the six tetraploid potato cultivars.**

**Figure S6. Number of SVs identified in un-transcribable haplotypes (UNTR hap) and transcribable haplotypes (TR hap).** *P* value is calculated using two-tailed Wilcoxon rank sum test.

**Figure S7. Graphical views of alignments of full-length transcripts against three haplotype sequences in the Spunta genome. a**, Transcript splice alignments of haplotype 1 (Hap1) indicate two perfectly matched transcripts with multiple isoforms. **b**, A single transcript spanning the full haplotype (Hap2), as shown by the splice alignments. **c**, No proper alignment of transcripts is detected in haplotype 3 (Hap3). Visualization of alignments is performed using Integrative Genomics Viewer version 2.3.97 [1].

**Figure S8. Amino-acid alignment surrounding two nonsynonymous substitutions, SNP126 and SNP387 among nine species. a**, Alignment surrounding SNP126. **b**, Alignment surrounding SNP387. *Solanum tuberosum* (Asn/His), potato sequences that carry the substitution changing Asparagine to Histidine. *Solanum tuberosum* (Ala/Thr), potato sequences that carry the substitution changing Alanine to Threonine.

**Figure S9. Illustration of the three allelic variants within the TPT domain of *GPT2.1*.** Presence frequency among starch varieties and other potatoes are shown in pie charts.

**Figure S1. Expression heat maps of starch related genes in roots, shoots, leaves, stolons, tubers, flowers and fruits of DM, in which expression levels are depicted as Log_2_(TPM+1).**

**Figure S2. Expression heat maps of SGA related genes in roots, shoots, leaves, stolons, tubers, flowers and fruits of DM, in which expression levels are depicted as Log_2_(TPM+1).**

**Figure S3. Number of three types of haplotypes that can be defined in the six potato cultivars for genes within or without regions of domestication sweeps.**

**Figure S4. Pair-wise alignment of amino acid sequences of the SQS gene in M6 (wild type, *S. chacoense*) and DM (domesticated type, *S. tuberosum*).** The transmembrane region is marked using a pink bar.

**Figure S5. Overview of percentage of transcribable and un-transcribable haplotypes in 75 starch-related genes among the six tetraploid potato cultivars.**

**Figure S6. Number of SVs identified in un-transcribable haplotypes (UNTR hap) and transcribable haplotypes (TR hap).** *P* value is calculated using two-tailed Wilcoxon rank sum test.

**Figure S7. Graphical views of alignments of full-length transcripts against three haplotype sequences in the Spunta genome. a**, Transcript splice alignments of haplotype 1 (Hap1) indicate two perfectly matched transcripts with multiple isoforms. **b**, A single transcript spanning the full haplotype (Hap2), as shown by the splice alignments. **c**, No proper alignment of transcripts is detected in haplotype 3 (Hap3). Visualization of alignments is performed using Integrative Genomics Viewer version 2.3.97 (ref. [1]).

**Figure S8. Amino-acid alignment surrounding two nonsynonymous substitutions, SNP126 and SNP387 among nine species. a**, Alignment surrounding SNP126. **b**, Alignment surrounding SNP387. *Solanum tuberosum* (Asn/His), potato sequences that carry the substitution changing Asparagine to Histidine. *Solanum tuberosum* (Ala/Thr), potato sequences that carry the substitution changing Alanine to Threonine.

**Figure S9. Illustration of the three allelic variants within the TPT domain of *GPT2.1*.** Presence frequency among starch varieties and other potatoes are shown in pie charts.

**References**

1. Thorvaldsdóttir H, Robinson JT, Mesirov JP: Integrative Genomics Viewer (IGV): high-performance genomics data visualization and exploration. Brief Bioinformatics 2013; 14(2):178-192.
